# Topical Timolol Inhibits Corneal Neovascularization in Rabbits

**Published:** 2017

**Authors:** Ali KASIRI, Mehdi Reza GHOMI, Mostafa FEGHHI, Fereydoun FARRAHI, Mohammad Sadegh MIRDEHGHAN, Hesam HEDAYATI

**Affiliations:** 1 Department of Ophthalmology, Ophthalmology Research Center, Jundishapur University of Medical Sciences, Ahvaz, Iran

**Keywords:** Cornea, Neovascularization, Vascular Endothelial Growth Factor (VEGF), Beta-blocker

## Abstract

Timolol is a non-selective beta-adrenergic antagonist that is similar to propranolol. The mechanism through which these drugs act on the regression of neovascularization is largely unknown. However, it is thought that the drugs may act through vascular endothelial growth factor signaling, vasoconstriction, and vascular endothelial cell apoptosis. The aim of this study was to determine the effect of timolol on corneal neovascularization in rabbits. Neovascularization was induced in the eyes of 20 rabbits. Next, the rabbits were divided into two groups: the timolol (experimental) group received eye drops containing timolol 0.5% twice per day; and the saline (control) group received saline drops twice per day for two weeks. After 7 days, the mean area of corneal neovascularization (presented as a percentage relative to baseline) was significantly lower in the timolol group than in the saline group (4.63 ± 4.61% versus 58.39 ± 6.31%, P < 0.001). After 2 weeks, the mean area of corneal neovascularization was 0.85 ± 1.33% in the timolol group and 1.73 ± 2.06% in the saline group (P = 0.315). After the first week of treatment, timolol significantly reduced the area of neovascularization compared to control. Timolol may increase the rate of recovery from corneal neovascularization.

## INTRODUCTION

The cornea is transparent due to a complex balance between cellular components and layers of the cornea [1]. Angiogenesis is the process of growing new blood vessels from existing vessel structures. Corneal angiogenesis occurs due to various pathological conditions and can have a number of adverse effects [2]. Corneal neovascularization may be secondary to chemical burns, ischemia, infection, trauma, and inflamation [3]. Vascular endothelial growth factor (VEGF) has an important role in the pathogenesis of neovascularization. Therefore, theoretically, any drugs interfering with VEGF signaling can be used to prevent the process of neovascularization. In previous studies, the anti-VEGF drug, bevacizumab, was shown to have positive effects on retinal and corneal neovascularization [4-8]. Recent studies have shown that angiogenesis is controlled by the adrenergic system via pro-angiogenic factors. This process is similar to that of the effect of norepinephrine on VEGF upregulation. Non-selective beta-adrenergic antagonists can produce electroretinogram (ERG) changes, which are associated with phosphorylation of insulin-like growth factor 1 receptor (IGF-1R) and regulation of VEGF [9]. In recent years, propranolol was introduced as the standard treatment for hemangioma [10]. However, its systemic administration, especially in children, is associated with adverse reactions such as bronchospasm, hypotension, hypoglycemia, and congestive heart failure [11, 12].

Timolol is a non-selective beta-adrenergic antagonist that is similar to propranolol. It is available as a topical solution with a concentration of 0.25% or 0.5% and a gel with a concentration of 1% or 0.5% for the treatment of glaucoma or ocular hypertension [13, 14]. The mechanism via which these drugs act on the regression of neovascularization is largely unknown. However, it is thought that the drugs may act via VEGF signaling, vasoconstriction, and vascular endothelial cell apoptosis [14-16]. Propranolol has no effect on the normal values of VEGF in the retina, but timolol reduces VEGF levels in oxygen-induced retinopathy [9]. In this study, we aimed to determine the effect of timolol on corneal neovascularization in rabbits. 

## MATERIALS AND METHODS

Twenty Iranian male rabbits, weighing 1500 to 1900 g, were used in this study. Animals were individually housed in standard cages in rooms at 22 ± 2°C. For adaptation to the new environment, animals were fed for 1 week with pellet food and water as needed. They were used for research and only one eye per animal was tested. The Research Council of Ahwaz Jundishapur University (code IORC-9504) approved this study. Under local anesthesia with tetracaine eye drops (Sinadaro Lab, Tehran, Iran), the right eyes of all rabbits were prepared as follows. 

**Figure 1:**
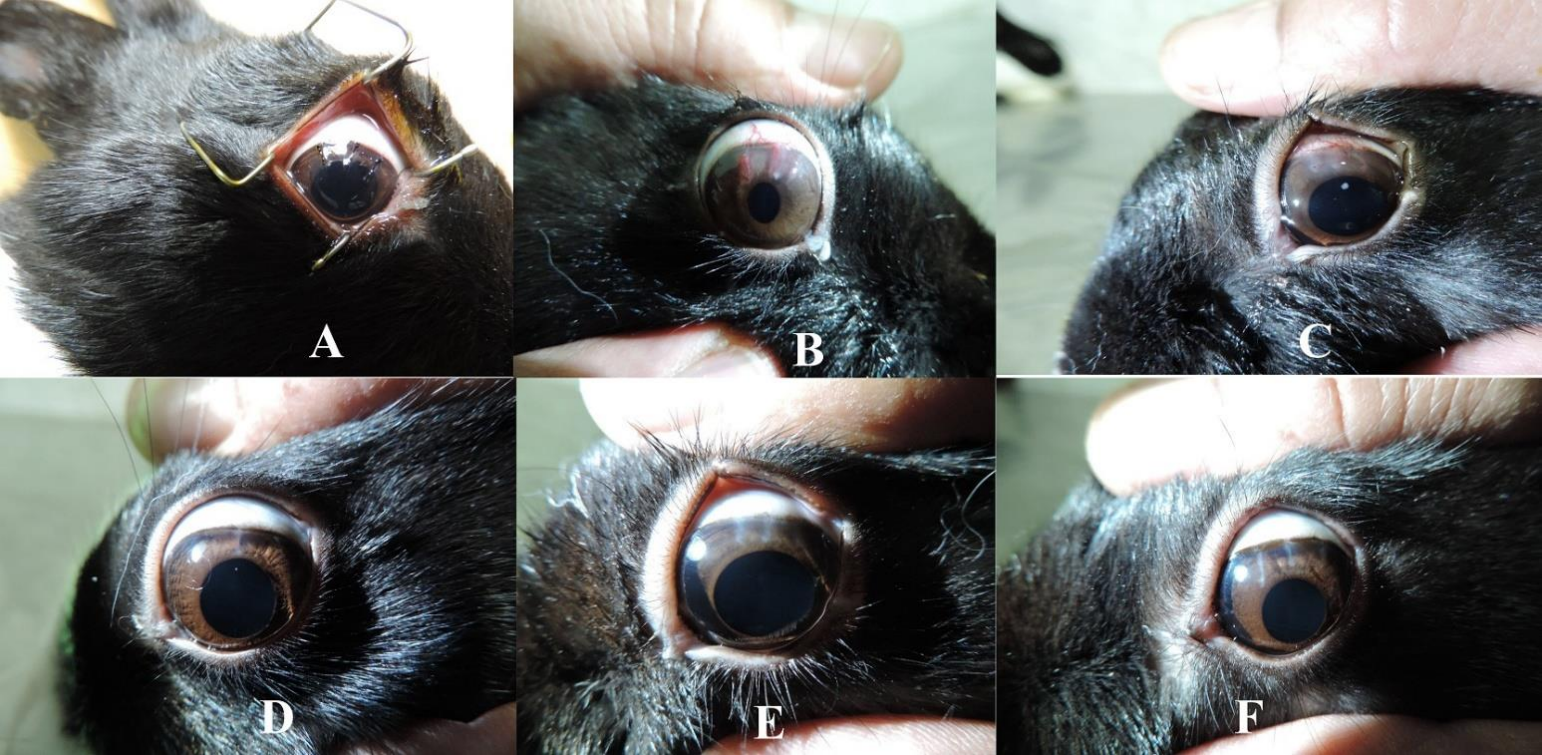
: A: Three 7-0 Silk Sutures; B: Corneal Neovascularization prior to Treatment; C: Week 1 in the Saline Group; D: Week 1 in the Timolol Group; E: Week 2 in the Saline Group; E and F: Week 2 in the Timolol Group

The eyelid was opened using an eye speculum and the eye was washed three times with 10 ml of normal saline. Then, three 7-0 silk sutures were placed radially at the pre-limbus in the upper region of the cornea at positions 10, 12, and 2 at a depth of 50% that of corneal thickness (Fig 1A).

The sutures were not buried. Ciprofloxacin eye drops (Sinadaro Lab. Tehran, Iran) were used to prevent infection and were continued for 2 weeks, three times a day. All sutures were placed in the same day by a single person. Two weeks after suture placement, all sutures were removed. Rabbits were randomly divided into two groups: the timolol (experimental) group received eye drops containing timolol 0.5% (Sinadaro Lab, Tehran, Iran) twice per day; and the saline (control) group received saline drops twice per day for two weeks. Immediately after removing the sutures, the first images of the cornea (representing early corneal neovascularization) were obtained (Fig 1B).

Corneal neovascularization was imaged using Canon cameras with a magnification of 32 times. Photos were analyzed using ImageJ analysis software, version 49/1 (ImageJ 1.49 for Windows) [17]. To determine the percentage of corneal neovascularization, the number of pixels covering areas of corneal neovascularization was determined before and 1 and 2 weeks after treatment. The area of neovascularization at baseline (before treatment) was defined as 100% (Fig 1C–F).

## RESULTS

**Figure 2:**
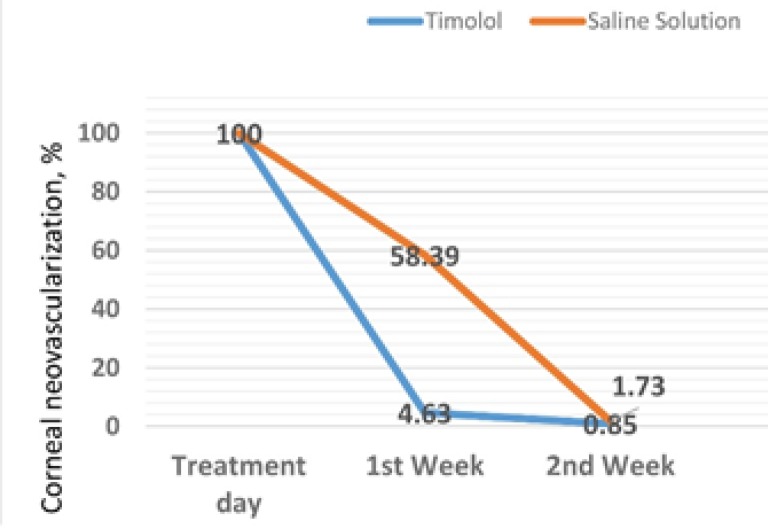
A: Changes in Corneal Neovascularization at 1 and 2 Weeks after Treatment in the Two Groups

After 1 week of treatment, the mean area of corneal neovascularization (presented as a percentage relative to baseline) was significantly lower in the timolol group than in the saline group (4.63 ± 4.61% versus 58.39 ± 6.31%, P < 0.001). After 2 weeks of treatment, the mean area of corneal neovascularization was 0.85 ± 1.33% in the timolol group and 1.73 ± 2.06% in the saline group (not significantly different, P = 0.315) (Tables 1 and 2, Figs 2). The changes in area of corneal neovascularization from baseline to 2 weeks after treatment are shown in Table 2. After 2 weeks of treatment, the changes were not significantly different between the two groups.

**Table 1:** The Area of Corneal Neovascularization at 1 and 2 Weeks after Treatment in the Two Groups

Group	N	Corneal neovascularization, % (mean ± standard deviation)	
		**Week 1**	**Week 2**
Timolol	10	61.63 ± 4.4	33.85 ± 1.0
Saline	10	31.39 ± 6.58	6.73 ± 2.1

**Table 2:** The Mean Change in Corneal Neovascularization after Two Weeks of Treatment Compared to the Beginning of Treatment in Two Study Groups

Group	N	Week 1 *	Week 2 *
Timolol	10	61.4 ± 95.37	33.1 ± 99.15
Saline	10	31.6 ± 41.61	6.2 ± 98.27

* Mean ± standard deviation

## DISCUSSION

This study investigated the effect of timolol on corneal neovascularization in rabbits. The results of this study suggest that compared to a saline control, timolol significantly reduced the area of corneal neovascularization at 1 week after treatment, but not at 2 weeks. The effect of bevacizumab on corneal neovascularization inhibition was demonstrated by Kim et al. in 2008 [18] and Oner et al. in 2012 [19]. In 2011, Ristori and colleagues reported the role of propranolol as a beta-adrenergic antagonist in decreasing the expression of VEGF and IGF-1, thus preventing the development of retinal neovascularization. Further, they revealed for the first time that blockage of the beta-adrenergic pathway protects against retinal angiogenesis and improves blood function in oxygen-induced retinopathy [9]. In 2014, Simavli and colleagues concluded that different doses of propranolol administered locally had a significant effect on corneal neovascularization in rats [20]. Earlier in 2008, Schwartz and colleagues showed that topically delivered anti-glaucoma drugs, namely latanoprost, dorzolamide, brimonidine, and timolol-malate may modify the normal angiogenic response in the rat cornea. Among the drugs used, prostaglandins revealed the most prominent pro-angiogenic consequence [21]. The present study was partly able to show the effect of timolol in the improvement of corneal neovascularization. However, the final effect of timolol was not significant due to spontaneous recovery in the control group. The limitation of this study was mainly lack of variation in timolol dosage.

## CONCLUSIONS

After the first week of treatment, timolol significantly reduced the area of neovascularization compared to control. Timolol increased the rate of recovery from corneal neovascularization. Topical timolol could be effective to prevent neovascularization. To confirm the effect of timolol, further studies with different dosages are required.
